# Supplementary material for: Comprehensive proteomic analysis of JC polyomavirus-infected human astrocytes and their extracellular vesicles
Source: Microbiol Spectr. 2023 Oct 10;11(6):e02751-23. doi: 10.1128/spectrum.02751-23 (PMC10714778; doi:10.1128/spectrum.02751-23)
Supplement: Supplemental material — List of abbreviations, Fig. S1 to S9, and Tables S1 to S5. [file spectrum.02751-23-s0001.docx]

**Supplementary material**

**Abbreviations**

| **ATM** | Ataxia-telangiectasia mutated protein |
| --- | --- |
| **ATR** | Ataxia telangiectasia and Rad3-related protein |
| **BML** | Bloom syndrome protein |
| **BMP1** | Bone morphogenetic protein 1 |
| **BRCA1** | Breast cancer gene 1 |
| **BST2** | Bone marrow stromal cell antigen 2 |
| **CCN** | Cyclin |
| **CDK** | Cyclin-dependent kinase |
| **CENPU** | Centromere protein U |
| **CEP55** | Centrosomal protein of 55 kDa |
| **CHEK2** | Checkpoint kinase 2 |
| **CKAP2** | Cytoskeleton-associated protein 2 |
| **CKAP2L** | Cytoskeleton Associated Protein 2 Like |
| **d.p.i** | Days post-infection |
| **DDR** | DNA damage response |
| **DDX** | DEAD-Box Helicase |
| **ER** | Endoplasmic reticulum |
| **EV** | Extracellular vesicle |
| **FANCD2** | Fanconi anemia D2 protein |
| **FANCG** | FA complementation group G |
| **GE** | Genome Equivalent |
| **GGT5** | Gamma-Glutamyltransferase 5 |
| **GINS2** | DNA Replication Complex GINS Protein PSF2 |
| **GO** | Gene ontology |
| **hiPSC** | Human induced pluripotent stem cell |
| **HNRNP** | Heterogeneous nuclear ribonucleoprotein |
| **IFA** | Immunofluorescence analysis |
| **IFIT1** | Interferon Induced Protein With Tetratricopeptide Repeats 1 |
| **IL-1β** | Interleukin-1 beta |
| **ILF3** | Interleukin enhancer-binding factor 3 |
| **IQGAP3** | IQ motif containing GTPase activating protein 3 |
| **ISG15** | Interferon-stimulated gene 15 |
| **JCPyV** | JC Polyomavirus |
| **KIF** | Kinesin-like protein |
| **KNSTRN** | Kinetochore localized astrin (SPAG5) binding protein |
| **LC-MS/MS** | Liquid chromatography-tandem mass spectrometry |
| **LT** | Large T-antigen |
| **MISEV** | Minimal information for studies of extracellular vesicles |
| **MS** | Multiple sclerosis |
| **MX1** | MX Dynamin Like GTPase 1 |
| **NCAPD3** | Condensin-2 complex subunit D3 |
| **NCAPH** | Non-SMC condensin I complex subunit H |
| **NDC80** | Kinetochore protein NDC80 homolog |
| **NUSAP1** | Nucleolar And Spindle Associated Protein 1 |
| **p53BP1** | p53-binding protein 1 |
| **PCNA** | Proliferating cell nuclear antigen |
| **PLK** | Polo-like kinase |
| **PML** | Progressive multifocal leukoencephalopathy |
| **PPI** | Protein-protein interaction |
| **RFC3** | Replication factor C subunit 3 |
| **SLC38A3** | Sodium-coupled neutral amino acid transporter 3 |
| **SRSF1** | Serine/arginine-rich splicing factor 1 |
| **ST** | Small t-antigen |
| **SV40** | Simian virus 40 |
| **TACC3** | Transforming acidic coiled-coil-containing protein 3 |
| **TEM** | Transmission electron microscopy |
| **TM9SF4** | Transmembrane 9 superfamily member 4 |
| **TMT** | Tandem mass tag |
| **TNF-α** | Tumor necrosis factor alpha |
| **TOP2A** | DNA topoisomerase 2 |
| **U2AF2** | Splicing factor U2AF 65 kDa subunit |
| **UBE2C** | Ubiquitin Conjugating Enzyme E2 C |
| **UHRF1** | Ubiquitin Like With PHD And Ring Finger Domains 1 |
| **yH2AX** | Phosphorylated Histone 2A variant |
| **ZWILCH** | Protein zwilch homolog |

**Supplementary figure legends**

**Supplementary Figure 1. Characterization of hiPSC-derived astrocytes used for the infection with JCPyV Mad-1.**

**A.** The expression profiles of 10 astrocytic and hiPSC markers were evaluated in hiPSC-derived astrocytes from two healthy donors used in this study (HC2 and HC3) and compared to that of hiPSCs and hiPSC-derived astrocytes characterized previously (17). The 10 markers are listed on the y axis and the cell type and donors are listed on the x-axis. Results are expressed as the Z-score of the -∆C_T_ (C_T_ of gene of interest – C_T_ of GAPDH).

**B, C.** Representative IFA images taken at 7 d.p.i. of JCPyV-infected and mock-infected astrocytes showing the co-localization of JCPyV capsid protein, VP1 (green), with astrocytic markers S100β (**B**) and EAAT1 (**C**) (red) (scale bar = 50 µm).

**Supplementary Figure 2. Focus-forming assay to determine number of infectious JCPyV particles in the supernatant of hiPSC-derived astrocytes at different timepoints post-infection.** Representative images of wells from 96-well plates comprising monolayers of SVG-A cells that were incubated with two-fold limiting dilutions of supernatants obtained from JCPyV-infected hiPSC-derived astrocytes at day 3, 7, 14 and 21 post-infection. At day 5, the SVG-A cells were fixed and stained for JCPyV LT (green) and nuclei counterstained with DAPI (blue). The number of focus-forming units (FFUs) were determined per ml of supernatant. The dilution factor is shown on the left and the day of infection that the supernatant was collected, on the top.

**Supplementary Figure 3. Transmission electron microscopy (TEM) of mock-infected astrocytes.**

**A, B.** Normal cellular morphologies are represented by two TEM images of mock-infected astrocytes at day 14 post-infection. Mock-infected cells comprised intact plasma membranes and cytoplasms (cyt) with the cell nuclei (nuc) devoid of virus particles and tubular structures.

**Supplementary Figure 4. Relative abundance of JCPyV early (LT and ST) and late (VP1 and VP2) proteins over time**. Cells were infected with JCPyV (red) or mock-infected (gray) as described in Fig. 1. At day 0, 3, 7, 14 and 21, the cell lysates were collected and analyzed by LS-MS/MS using a TMT labeling approach. Each dot on the graph represents an experimental replicate and the line the line represents the median. All graphs: n = 3 independent infections performed per readout, with each dot on the graph representing an individual experiment (in red: JCPyV; In grey: mock) and the line links the median value of each condition. The effect of infection over time (D0 vs other timepoints) was tested using a two-way ANOVA followed by Sidak`s multiple comparison test. Statistical significance of data: *p < 0.05; ** p<0.01; ***p < 0.001; ****p < 0.0001.

**Supplementary Figure 5. Selection of hundred highest-ranking host proteins in JCPyV-infected conditions.** Cells were infected with JCPyV or mock-infected as described in Fig. 1. Representative scatter plot showing quantified proteins in JCPyV conditions as compared to mock-infected control at day 21 of infection. the Log_2_(fold-change) of the quantified proteins are represented on the x-axis (JCPyV/ Mock) and the corresponding -Log_10_(P-value) on the y-axis. Significantly (FDR ≤ 0.05) dysregulated proteins are shown in color, with upregulated proteins shown in red and downregulated proteins shown in blue. The hundred highest-ranked proteins (yellow dots), according to the product of |-log_10_(P-value) * log_2_(fold-change)|, were selected for downstream analysis (see Fig. 5).

**Supplementary Figure 6. Immunofluorescence analysis (IFA) of DNA damage response (DDR) proteins in mock-infected astrocytes.**

Representative images taken at 7 d.p.i. of mock-infected astrocytes showing a lack of formation of nuclear foci (yH2AX, BRCA1, FANCD2, p53BP1) and no upregulation of DNA damage checkpoint proteins (ATM, CHEK2, PLK). JCPyV LT is stained in red and VP1 in green (scale bar = 50 µm).

**Supplementary Figure 7. Quality check of extracellular vesicles (EVs) isolated from JCPyV-infected or cytokine-stimulated astrocytes.**

**A, B.** Violin plots representing the abundances (riBAQ score) of EV-associated proteins (categories 1A, 1B, 2A, 2B) as compared to major contaminant proteins typically co-isolated in EV preparations (categories 3A, 3B), according to MISIEV2018 specifications. EV-associated proteins were significantly enriched in all conditions analyzed (**A:** JCPyV vs mock-infected; **B:** cytokine-stimulated vs resting) as compared to contaminant proteins. The significance was determined by using a nonparametric paired Wilcoxon test: * p<0.05; ****, p <0.0001.

**Supplementary Figure 8. Association of JCPyV VP1 protein with EVs from hiPSC-derived astrocytes.** EVs were isolated from JCPyV (red) or mock-infected (grey) astrocytes and subjected to density gradient ultracentrifugation (DGUC). As a control, the same procedure was done for JCPyV free virus (green). Collected fractions (F) comprising densities ranging between 1.07 to 1.21 g/ml were analysed by western blot for the presence of JCPyV VP1 and EV marker, CD81.

**Supplementary Figure 9. Selection of the hundred highest-ranked host proteins in EVs from JCPyV-infected or cytokine stimulated conditions.** Human iPSC-derived astrocytes were infected with a MOI of 0.07 JCPyV Mad-1 or stimulated with 10ng/ml TNFα and 10ng/ml IL-1β. As the negative control for each condition, the cells were either mock-infected or left resting, respectively.

**A, B.** Representative scatter plots showing quantified proteins in JCPyV-infected (**A**) or cytokine-stimulated (**B**) conditions at day 14 as compared to mock-infected, or resting controls, respectively. The Log_2_(fold-change) of the quantified proteins are represented on the x-axis and the corresponding -Log_10_(P-value) on the y-axis. Significantly (FDR ≤ 0.05) dysregulated proteins are shown in color, with upregulated proteins shown in red and downregulated proteins shown in blue. The hundred highest-ranked proteins in each condition (yellow dots), according to the product of |-log_10_(P-value) * log_2_(fold-change)|, were selected for downstream analysis (see Fig. 8).

**Supplementary figures**

**Supplementary Figure 1.**

**
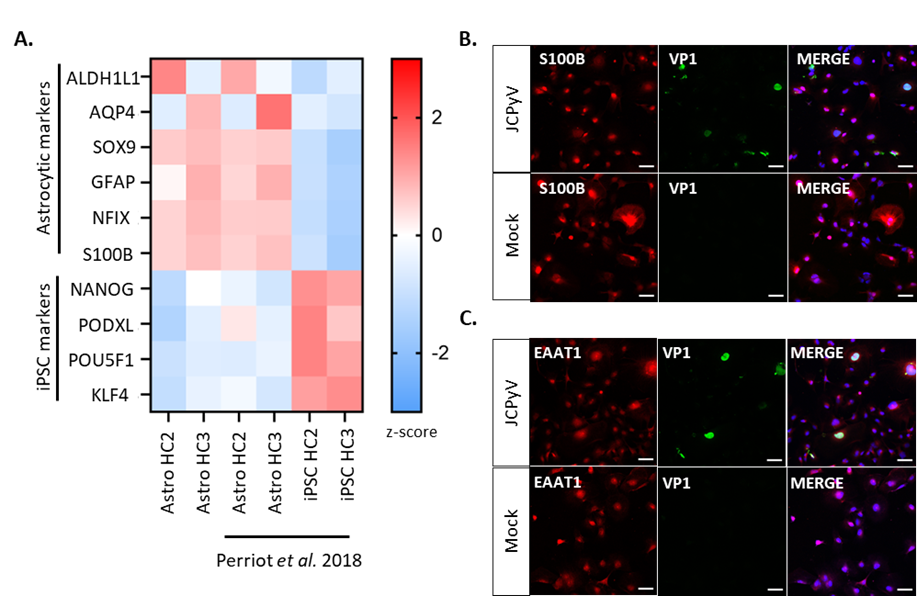
**

**Supplementary Figure 2.**

**
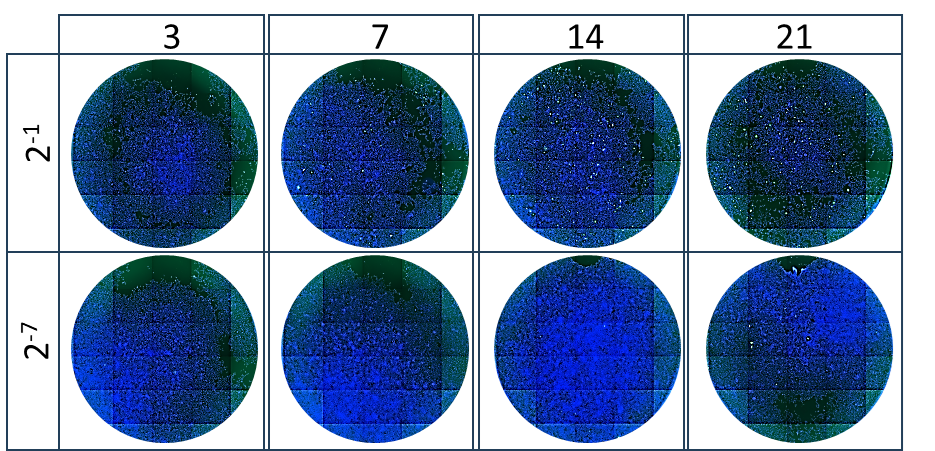
**

**Supplementary Figure 3.**


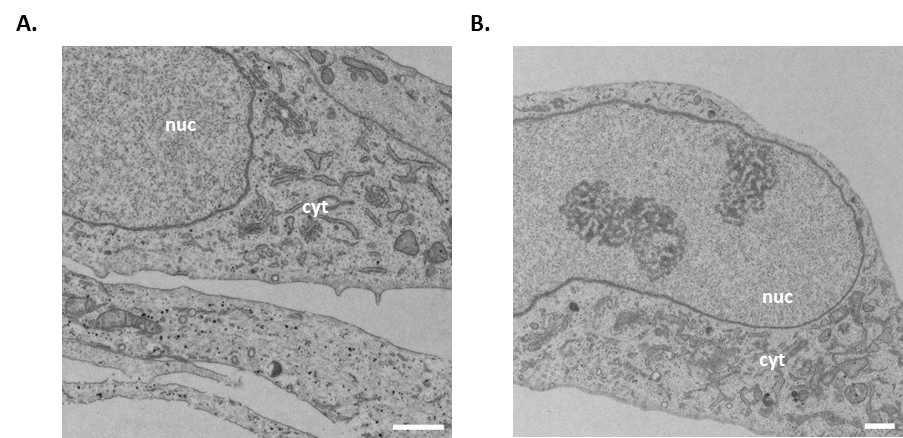


**Supplementary Figure 4.**

**Supplementary Figure 5.**


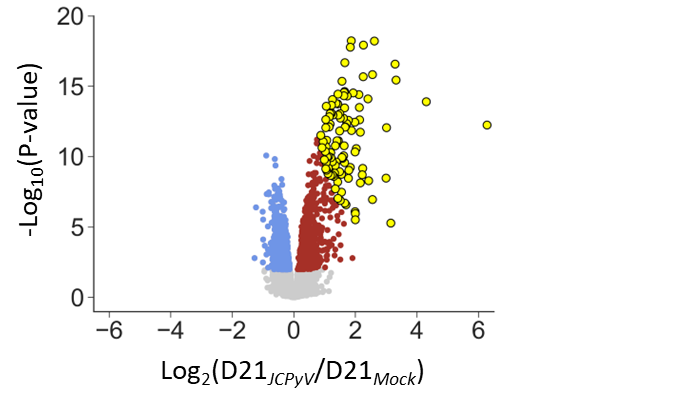


**Supplementary Figure 6.**


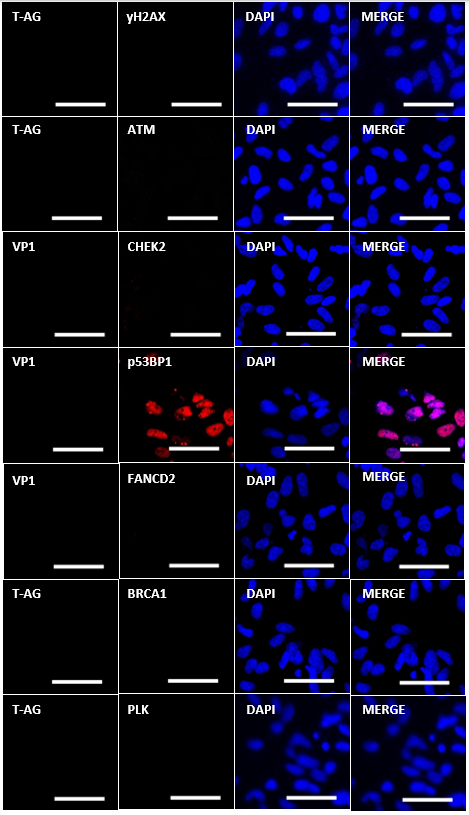


**Supplementary Figure 7.**


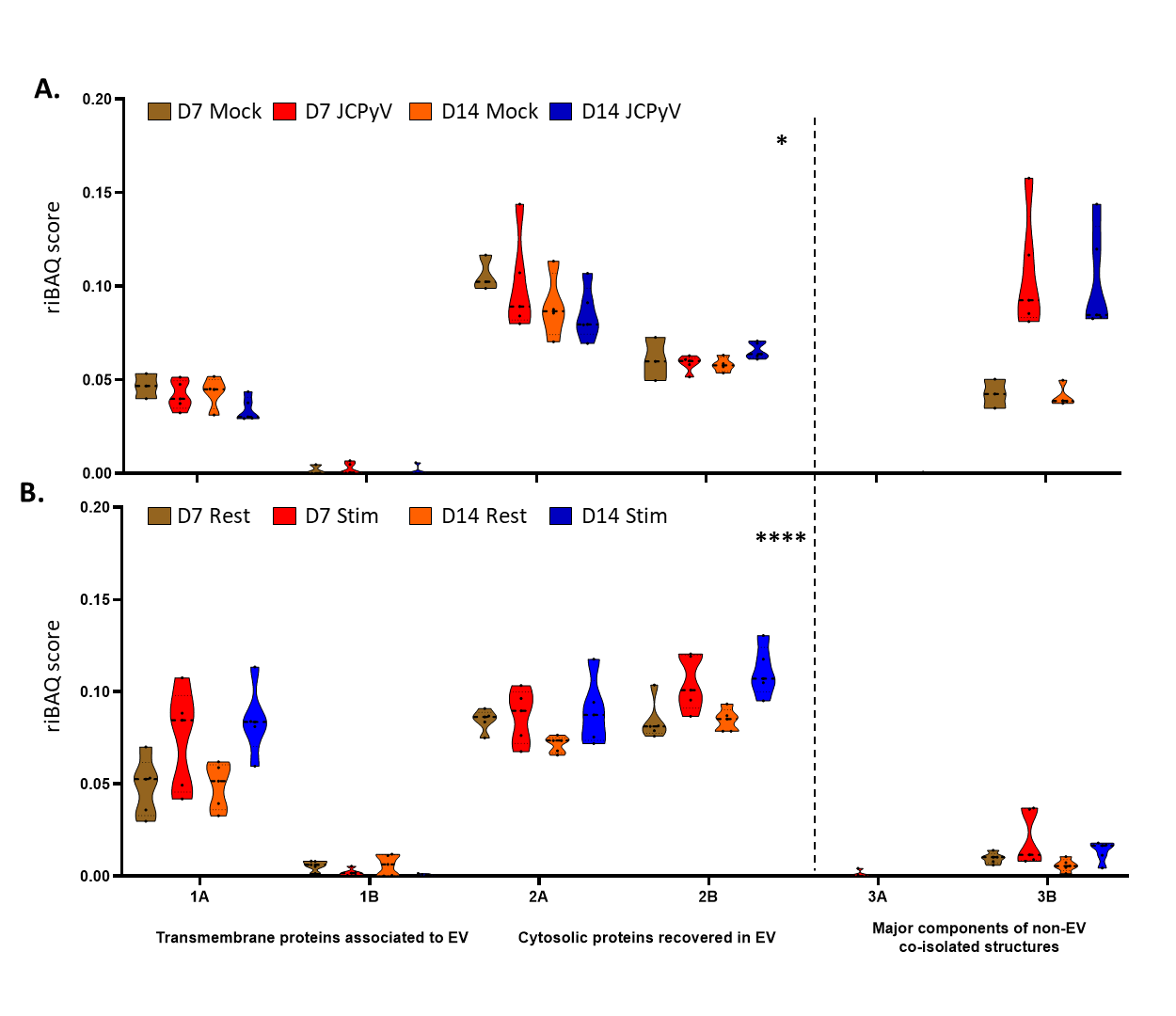


**Supplementary Figure 8.**


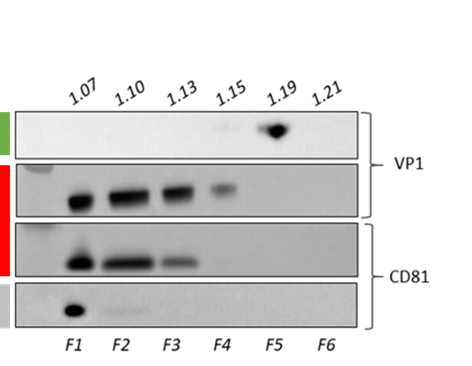


**Supplementary Figure 9.**

**
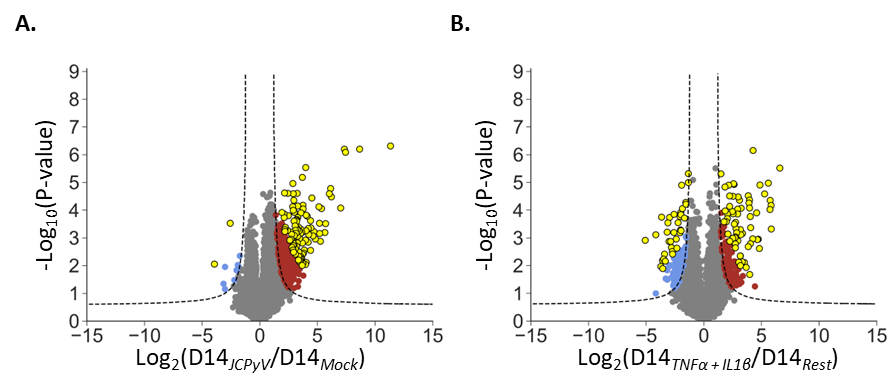
**

**Supplementary Table 1. Gene Ontology (GO) enrichment analysis of JCPyV-infected astrocytes.** The ten highest-ranked GO terms are indicated with the corresponding FDR value, number of enriched genes and number of background genes in that pathway. The names of the genes are shown in the final column followed by fold-change (underlined) of the gene in the infected conditions as compared to the mock-infected control and corresponding P-value (italic) of the fold-change.

| Term name | Description | FDR | Number of genes | Number of Background genes | Gene names |
| --- | --- | --- | --- | --- | --- |
| GO:0007049 | Cell cycle | 2,40E-68 | 79 | 1313 | RFC2 (1.22, *13.65*); KIF22 (1.02, *10.37*); TRIP13 (1.07, *11.16*); MCM5 (1.66, *12.10*); MSH6 (1.22, *13.09*); NCAPH (1.19, *8.66*); KNSTRN (1.51, *6.90*); NCAPG (1.25, *14.05*); CCNB1 (2.99, *8.47*); KIF23 (1.66, *6.70*); KIF11 (2.62, *18.21*); NDC80 (1.55, *8.74*); MCM4 (1.72, *12.35*); PRR11 (2.12, *14.42*); LIG1 (1.87, *18.23*); MCM6 (1.62, *14.33*); MCM2 (1.66, *16.68*); ANLN (2.26, *17.92*); CDK2 (1.20, *10.29*); CCNA2 (1.87, *11.85*); SPC25 (1.48, *8.93*); CENPH (1.77, *8.45*); SMC2 (1.65, *14.63*); FANCD2 (1.56, *15.37*); CCNB2 (2.22, *8.71*); MAD2L1 (1.15, *12.84*); PLK1 (1.37, *9.63*); TPX2 (1.62, *10.06*); RRM1 (1.05, *13.58*); BIRC5 (1.12, *9.19*); FEN1 (1.27, *12.96*); MCM7 (1.73, *14.31*); FANCI (1.63, *14.57*); ZWILCH (1.04, *9.13*); CHAF1B (1.65, *13.47*); POLE (1.05, *9.57*); KIF15 (1.42, *13.75*); NCAPD2 (1.14, *12.11*); TACC3 (1.57, *9.95*); KNL1 (1.29, *8.60*); BLM (2.55, *6.95*); UBE2C (1.50, *13.06*); SMC4 (1.35, *10.77*); RRM2 (1.98, *12.46*); IQGAP3 (0.91, *10.64*); CENPF (1.45, *11.14*); MKI67 (2.26, *15.69*); CEP55 (1.55, *7.47*); ZWINT (1.65, *9.58*); KIF4A (1.40, *11.13*); CKS2 (1.99, *10.35*); CKAP2 (3.01, *12.07*); PCNA (1.03, *12.06*); RFC3 (1.43, *14.45*); CIT (1.18, *13.07*); ECT2 (2.03, *10.58*); HELLS (1.44, *12.96*); PRC1 (2.43, *8.30*); KIF20A (1.43, *8.21*); CDK1 (1.93, *14.54*); CENPK (1.25, *8.70*); WDR62 (1.25, *9.65*); NCAPG2 (1.84, *17.78*); KIFC1 (2.41, *14.10*); TOP2B (0.95, *11.06*); RACGAP1 (1.79, *9.03*); CDC45 (1.34, *9.37*); POLD1 (1.05, *10.70*); RFC5 (1.37, *13.78*); TOP2A (3.32, *15.44*); BRCA1 (1.99, *6.09*); PBK (2.55, *15.84*); NCAPD3 (0.99, *9.78*); TIMELESS (1.11, *8.80*); NUSAP1 (2.13, *12.62*); KIF18B (2.23, *9.16*); UHRF1 (1.03, *12.64*); MCM3 (1.61, *12.74*); FAM83D (1.43, *7.03*) |
| GO:0030261 | Chromosome condensation | 6,11E-13 | 11 | 42 | NCAPH (1.19, *8.66*); NCAPG (1.25, *14.05*); CCNB1 (2.99, *8.47*); SMC2 (1.65, *14.63*); NCAPD2 (1.14, *12.11*); SMC4 (1.35, *10.77*); CDK1 (1.93, *14.54*); NCAPG2 (1.84, *17.78*); TOP2A (3.32, *15.44*); NCAPD3 (0.99, *9.78*); NUSAP1 (2.13, *12.62*) |
| GO:0000727 | DSB repair via break-induced replication | 6,24E-12 | 8 | 11 | MCM5 (1.66, *12.10*); GINS2 (1.48, *11.82*); MCM4 (1.72, *12.35*); MCM6 (1.62, *14.33*); MCM2 (1.66, *16.68*); MCM7 (1.73, *14.31*); CDC45 (1.34, *9.37*); MCM3 (1.61, *12.74*) |
| GO:0033045 | Regulation of sister chromatid segregation | 6,11E-09 | 10 | 83 | TRIP13 (1.07, *11.16*); CCNB1 (2.99, *8.47*); NDC80 (1.55, *8.74*); MAD2L1 (1.15, *12.84*); PLK1 (1.37, *9.63*); BIRC5 (1.12, *9.19*); FEN1 (1.27, *12.96*); TACC3 (1.57, *9.95*); UBE2C (1.50, *13.06*); CENPF (1.45, *11.14*) |
| GO:0006297 | Nucleotide-excision repair, DNA gap filling | 1,81E-08 | 7 | 23 | RFC2 (1.22, *13.65*); LIG1 (1.87, *18.23*); POLE (1.05, *9.57*); PCNA (1.03, *12.06*); RFC3 (1.43, *14.45*); POLD1 (1.05, *10.70*); RFC5 (1.37, *13.78*) |
| GO:0090307 | Mitotic spindle assembly | 2,33E-08 | 8 | 43 | KIF23 (1.66, *6.70*); KIF11 (2.62, *18.21*); TPX2 (1.62, *10.06*); BIRC5 (1.12, *9.19*); KIF4A (1.40, *11.13*); PRC1 (2.43, *8.30*); KIFC1 (2.41, *14.10*); RACGAP1 (1.79, *9.03*) |
| GO:0051383 | Kinetochore organization | 2,22E-07 | 6 | 18 | NDC80 (1.55, *8.74*); CENPH (1.77, *8.45*); SMC2 (1.65, *14.63*); SMC4 (1.35, *10.77*); CENPF (1.45, *11.14*); CENPK (1.25, *8.70*) |
| GO:0000077 | DNA damage checkpoint | 6,99E-06 | 9 | 140 | CCNB1 (2.99, *8.47*); CDK2 (1.20, *10.29*); FANCD2 (1.56, *15.37*); PLK1 (1.37, *9.63*); BLM (2.55, *6.95*); DTL (2.16, *11.74*); PCNA (1.03, *12.06*); CDK1 (1.93, *14.54*); BRCA1 (1.99, *6.09*) |
| GO:0051988 | Attachment of spindle microtubules to kinetochore | 1,10E-04 | 4 | 13 | KNSTRN (1.51, *6.90*); CCNB1 (2.99, *8.47*); ECT2 (2.03, *10.58*); RACGAP1 (1.79, *9.03*) |
| GO:0061982 | Meiosis I cell cycle process | 2,00E-04 | 7 | 114 | TRIP13 (1.07, *11.16*); MSH6 (1.22, *13.09*); FANCD2 (1.56, *15.37*); PLK1 (1.37, *9.63*); CKS2 (1.99, *10.35*); TOP2B (0.95, *11.06*); TOP2A (3.32, *15.44*) |

**Supplementary Table 2. Gene Ontology (GO) enrichment analysis of EVs from JCPyV-infected astrocytes.** The ten highest-ranked GO terms are indicated with the corresponding FDR value, number of enriched genes and number of background genes in that pathway. The names of the genes are shown in the final column followed by fold-change (underlined) of the gene in the infected conditions as compared to the mock-infected control and corresponding P-value (italic) of the fold-change.

| Term name | Description | FDR | Number of genes | Number of Background genes | Gene names |
| --- | --- | --- | --- | --- | --- |
| GO:0000398 | mRNA splicing, via spliceosome | 2,63E-24 | 29 | 294 | FRG1 (2.87, *2.89*); SRSF9 (3.28, *3.06*); SRSF6 (3.38, *2.34*); RALY (3.85, *2.57*); SNRNP40 (3.35, *2.20*); PRPF6 (3.37, *3.38*); EIF4A3 (3.38, *2.49*); GEMIN7 (1.91, *3.92*); HNRNPU (3.09, *2.42*); U2AF1L5 (3.19, *2.70*); LUC7L (3.28, *3.74*); TRA2A (2.84, *2.62*); U2AF2 (2.23, *3.38*); HNRNPA0 (3.97, *2.04*); SNRNP200 (3.46, *2.14*); SRSF7 (4.12, *3.78*); DDX20 (2.97, *3.61*); SRSF4 (3.67, *2.01*); SRSF2 (3.06, *3.44*); PRPF40A (3.08, *2.61*); DDX46 (4.25, *3.12*); SRSF10 (3.29, *4.15*); HNRNPC (2.61, *2.92*); SRSF5 (4.20, *2.85*); RNPS1 (4.02, *2.11*); PRPF8 (3.79, *2.45*); SNRNP70 (3.26, *3.29*); POLR2A (3.70, *2.82*); SRRT (3.58, *3.31*) |
| GO:0007049 | Cell cycle | 4,54E-20 | 42 | 1313 | MCM5 (4.81, *2.96*); NCAPH (4.61, *3.07*); HAUS8 (2.11, *3.73*); NASP (2.90, *3.99*); CCNB1 (3.98, *5.54*); KIF11 (6.02, *4.59*); NDC80 (3.96, *2.92*); MCM4 (5.36, *4.06*); MCM6 (3.74, *2.08*); MCM2 (4.43, *3.36*); SPC25 (3.21, *3.66*); HNRNPU (3.09, *2.42*); SMC2 (4.51, *4.44);* PSME3 (2.93, *2.48*); RRM1 (4.40, *2.55*); FEN1 (5.71, *3.52*); MCM7 (5.26, *2.88*); CCAR2 (4.01, *3.26*); CKS1B (2.17, *3.30*); PRKDC (5.20, *3.16*); TYMS (3.69, *5.18*); SMC1A (3.45, *2.98*); NCAPD2 (4.40, *3.62*); TACC3 (2.41, *3.27*); PPM1G (2.86, *2.83*); SMC4 (6.21, *4.48*); MCMBP (2.89, *4.59*); SMC3 (3.70, *2.40*); CENPF (3.10, *3.28*); MKI67 (3.54, *3.16*); KIF2C (5.12, *4.12*); RCC1 (3.65, *2.25*); SRSF2 (3.06, *3.44*); CIT (2.89, *2.56*); CDK1 (2.49, *3.80*); PRPF40A (3.08, *2.61*); DHFR (4.02, *3.82*); WEE1 (2.68, *3.21*); TOP2A (7.02, *4.08*); PBK (3.75, *4.04*); SPC24 (3.40, *3.25*); MCM3 (6.14, *4.78*) |
| GO:0006267 | Pre-replicative complex assembly | 7,57E-09 | 6 | 7 | MCM5 (4.81, *2.96*); MCM4 (5.36, *4.06*); MCM6 (3.74, *2.08*); MCM2 (4.43, *3.36*); MCM7 (5.26, *2.88*); MCM3 (6.14, *4.78*) |
| GO:0010032 | Meiotic chromosome condensation | 2,46E-05 | 4 | 7 | NCAPH (4.61, *3.07*); SMC2 (4.51, *4.44*); NCAPD2 (4.40, *3.62*); SMC4 (6.21, *4.48*) |
| GO:0000244 | Spliceosomal tri-snRNP complex assembly | 1,60E-04 | 4 | 13 | PRPF6 (3.37, *3.38*); DDX20 (2.97, *3.61*); SRSF10 (3.29, *4.15*); PRPF8 (3.79, *2.45*) |
| GO:0033045 | Regulation of sister chromatid segregation | 6,10E-04 | 6 | 83 | CCNB1 (3.98, *5.54*); NDC80 (3.96, *2.92*); HNRNPU (3.09, *2.42*); FEN1 (5.71, *3.52*); TACC3 (2.41, *3.27*); CENPF (3.10, *3.28*) |
| GO:0006364 | rRNA processing | 0,0016 | 8 | 212 | FRG1 (2.87, *2.89*); EIF4A3 (3.38, *2.49*); TSR1 (3.22, *3.35*); PRKDC (5.20, *3.16*); EXOSC4 (3.56, *3.90*); DDX21 (4.69, *3.23*); MRTO4 (3.73, *4.18*); ERI1 (2.96, *4.36*) |
| GO:0006325 | Chromatin organization | 0,007 | 13 | 713 | SUPT16H (5.65, *3.19*); NASP (2.90, *3.99*); CCNB1 (3.98, *5.54*); HAT1 (3.20, *3.59*); MCM2 (4.43, *3.36*); SMARCC2 (2.90, *2.72*); HNRNPU (3.09, *2.42*); CHD4 (4.21, *3.34*); CDK1 (2.49, *3.80*); DEK (3.52, *2.17*); HDAC2 (3.64, *3.19*); HNRNPC (2.61, *2.92*); SAFB (2.10, *3.86*) |
| GO:0051225 | Spindle assembly | 0,0097 | 5 | 88 | HAUS8 (2.11, *3.73*); KIF11 (6.02, *4.59*); SMC1A (3.45, *2.98*); SMC3 (3.70, *2.40*); RCC1 (3.65, *2.25*) |
| GO:0009263 | Deoxyribonucleotide biosynthetic process | 0,0099 | 3 | 16 | RRM1 (4.40, *2.55*); TYMS (3.69, *5.18*); DUT (3.56, *2.32*) |

**Supplementary Table 3. Gene Ontology (GO) enrichment analysis of EVs from cytokine-stimulated (TNFα+IL-1β) astrocytes.** The ten highest-ranked GO terms are indicated with the corresponding FDR value, number of enriched genes and number of background genes in that pathway. The names of the genes are shown in the final column followed by fold-change (underlined) of the gene in the stimulated conditions as compared to the resting control and corresponding P-value (italic) of the fold-change.

| Term name | Description | FDR | Number of genes | Number of Background genes | Gene names |
| --- | --- | --- | --- | --- | --- |
| GO:0019221 | Cytokine-mediated signaling pathway | 8.62E-14 | 28 | 678 | PSME2 (2.17, *4.90*); NFKB1 (3.86, *4.52*); OAS3 (4.87, *4.91*); CD70 (2.94, *4.03*); BST2 (5.84, *4.17*); TRIM21 (2.76, *3.56*); ICAM1 (3.97, *4.36*); RQCD1 (3.15, *2.35*); VCAM1 (2.98, *4.48*); OAS2 (4.72, *2.95*); PSMB10 (4.05, *2.79*); STAT1 (1.95, *3.58*); GBP2 (3.92, *4.26*); GBP1 (1.49, *5.31*); IFIT1 (5.73, *4.02*); IFIT3 (5.28, *4.59*); IFIT2 (3.35, *3.16*); TRAF1 (3.53, *2.10*); PSMB9 (2.59, *4.96*); PSMB8 (2.25, *4.14*); HLA-C (2.05, *3.70*); PSME1 (1.83, *4.83*); HLA-A (2.49, *3.95*); MX1 (6.60, *5.51*); HLA-B (2.63, *4.92*); SOD2 (5.59, *4.97*); B2M (1.85, *3.97*); HNRNPDL (4.82, *2.62*) |
| GO:0019885 | Antigen processing and presentation via MHC class I | 2.46E-06 | 5 | 10 | HLA-C (2.05, *3.70*); HLA-A (2.49, *3.95*); HLA-B (2.63, *4.92*); TAPBP (4.03, *3.77*); B2M (1.85, *3.97*) |
| GO:1903311 | Regulation of mRNA metabolic process | 6.35E-06 | 13 | 338 | PSME2 (2.17, *4.90*); HNRNPL (3.17, *3.30*); DDX5 (2.51, *2.99*); U2AF2 (2.71, *3.01*); HNRNPD (1.75, *3.58*); HNRNPM (2.10, *3.14*); PTBP1 (2.55, *3.87*); PSMB10 (4.05, *2.79*); PSMB9 (2.59, *4.96*); PSMB8 (2.25, *4.14*); PSME1 (1.83, *4.83*); DDX17 (3.17, *2.35*); ELAVL1 (2.75, *3.03*) |
| GO:0032479 | Regulation of type I interferon production | 8.56E-05 | 8 | 130 | RELB (2.94, *3.86*); NFKB1 (3.86, *4.52*); TRIM21 (2.76, *3.56*); UBA7 (4.27, *6.15*); STAT1 (1.95, *3.58*); NFKB2 (4.58, *3.90*); DDX58 (5.76, *4.35*); TNFAIP3 (3.85, *2.69*) |
| GO:0033993 | Response to lipid | 2.60E-04 | 17 | 858 | MMP15 (-2.88, *2.87*); DDX5 (2.51, *2.99*); NFKB1 (3.86, *4.52*); ICAM1 (3.97, *4.36*); NOTCH1 (-1.55, *4.21*); GJA1 (-2.98, *3.74*); ITGA2 (2.05, *3.87*); HNRNPD (1.75, *3.58*); ATP1A2 (-3.35, *2.16*); NFKB2 (4.58, *3.90*); CD274 (4.64, *2.93*); DDX17 (3.17, *2.35*); GGT5 (5.86, *3.32*); CRYAB (-3.57, *1.90*); DAG1 (-1.93, *4.90*); B2M (1.85, *3.97*); TNFAIP3 (3.85, *2.69*) |
| GO:0007155 | Cell adhesion | 6.10E-04 | 17 | 925 | CHL1 (-2.38, *2.96*); CNTNAP1 (3.17, *2.00*); ICAM1 (3.97, *4.36*); EPHA4 (-2.23, *3.55*); VCAM1 (2.98, *4.48*); ITGA2 (2.05, *3.87*); COL14A1 (-3.73, *1.97*); CTNND2 (-3.48, *2.23*); NLGN3 (-2.71, *2.41*); PTPRF (-2.81, *2.65*); GPC4 (-1.99, *3.91*); TTYH1 (-3.17, *3.21*); CADM2 (-2.73, *2.51*); CTNNA2 (-1.99, *3.84*); TENM3 (-1.56, *4.28*); TENM2 (-2.01, *3.29*); DAG1 (-1.93, *4.90*) |
| GO:2001014 | Regulation of skeletal muscle cell differentiation | 0.0114 | 3 | 19 | DDX5 (2.51, *2.99*); GPC1 (-1.97, *3.26*); DDX17 (3.17, *2.35*) |
| GO:0098942 | Retrograde trans-synaptic signaling | 0.0153 | 2 | 3 | TENM2 (-2.01, *3.29*); DAG1 (-1.93, *4.90*) |
| GO:0035455 | Response to interferon-alpha | 0.0175 | 3 | 23 | BST2 (5.84, *4.17*); IFIT3 (5.28, *4.59*); IFIT2 (3.35, *3.16*) |
| GO:1903902 | Positive regulation of viral life cycle | 0.0199 | 4 | 62 | TRIM21 (2.76, *3.56*); VPS37B (-1.34, *4.99*); NOTCH1 (-1.55, *4.21*); IFIT1 (5.73, *4.02*) |

**Supplementary Table 4.** List of primers used for reverse transcription quantitative PCR (RT-qPCR).

| **Gene name** | **Forward** | **Reverse** |
| --- | --- | --- |
| ALDH1L1 | GCTGACTGTGACCTCAACAA | GCTGCAATGCAATTCTCTCC |
| AQP4 | CAGGAATCCTCTATCTGGTCACA | GAGACCATGACCAGCGGTAA |
| SOX9 | AACGCCGAGCTCAGCAA | CGCTTCTCGCTCTCGTTCA |
| GFAP | GCCAGTTGCAGTCCTTGAC | GCGCATCTGCCTCTCCA |
| NFIX | CAAGGAGATGCGGACATCAAAC | ACCCCGGAAGTCACAAAACA |
| S100B | GCAGCAAGGAGACCAGGAA | CCACCATGGCCTTCTCCA |
| NANOG | TGCAGAGAAGAGTGTCGCAAA | GCTGGGTGGAAGAGAACACA |
| PODXL | GATGTGTACGAGCGGCTGAA | TGGTCCCCTAGCTTCATGTCA |
| POUDF1 | GGGGACCAGTGTCCTTTCC | GGGAAAGGGACCGAGGAGTA |
| KLF4 | CTGCGGCAAAACCTACACAA | CGTCCCAGTCACAGTGGTAA |

**Supplementary Table 5.** Quality check of EVs based on MISEV2018 specifications (30). Proteins categorized with a "*" in the study were manually extended using the Gene Ontology website.

| 1A- NON TISSUE SPECIFIC | 1B- CELL / TISSUE SPECIFIC | 2A- WITH LIPID OR MEMBRANE PROTEIN BINDING ABILITY | 2B- PROMISCUOUS INCORPORATION IN EVs | 3A- LIPOPROTEINS | 3B- PROTEIN AND PROTEIN / NUCLEIC ACID AGGREGATES |
| --- | --- | --- | --- | --- | --- |
| ADAM10 | A4 | ANXA1 | ACTA1 | ALB | DAP3 |
| BSG | ABCC1 | ANXA10 | ACTA2 | APOA1 | MRPL1 |
| CD47 | AChE-E | ANXA11 | ACTB | APOA2 | MRPL10 |
| CD55 | AChE-S | ANXA13 | ACTBL2 | APOB | MRPL11 |
| CD59 | APP | ANXA2 | ACTC1 | APOB100 | MRPL12 |
| CD63 | CD14 | ANXA2P2 | ACTG1 |  | MRPL13 |
| CD81 | CD300A | ANXA2R | ACTG2 |  | MRPL14 |
| CD82 | CD300C | ANXA3 | ACTL10 |  | MRPL15 |
| GNA | CD300E | ANXA4 | ACTL6A |  | MRPL16 |
| GNA11 | CD300H | ANXA5 | ACTL6B |  | MRPL17 |
| GNA12 | CD300LB | ANXA6 | ACTL7A |  | MRPL18 |
| GNA13 | CD300LD | ANXA7 | ACTL7B |  | MRPL19 |
| GNA14 | CD300LF | ANXA8 | ACTL8 |  | MRPL2 |
| GNA15 | CD300LG | ANXA8L1 | ACTL9 |  | MRPL20 |
| GNAI1 | CD302 | ANXA9 | ACTN1 |  | MRPL21 |
| GNAI2 | CD320 | ARF6 | ACTN2 |  | MRPL22 |
| GNAI3 | CD33 | ARRDC1 | ACTN3 |  | MRPL23 |
| GNAL | CD34 | CAV1 | ACTN4 |  | MRPL24 |
| GNAO1 | CD36 | CAV2 | ACTR10 |  | MRPL27 |
| GNAQ | CD37 | CAV3 | ACTR1A |  | MRPL28 |
| GNAS | CD38 | CAVIN1 | ACTR2 |  | MRPL3 |
| GNAT1 | CD3D | CAVIN2 | ACTR3 |  | MRPL30 |
| GNAT2 | CD3E | CAVIN3 | ACTR3B |  | MRPL32 |
| GNAT3 | CD3G | CAVIN4 | ACTR3C |  | MRPL33 |
| GNAZ | CD53 | CHMP1A | ACTR5 |  | MRPL34 |
| H2-D | CD9 | CHMP1B | ACTR6 |  | MRPL35 |
| H2-K | EPCAM | CHMP2A | ACTR8 |  | MRPL36 |
| H2-Q | ERBB2 | CHMP2B | ACTRT1 |  | MRPL37 |
| HLA-A | GP9 | CHMP3 | ACTRT2 |  | MRPL38 |
| HLA-B | GYPA | CHMP4A | ACTRT3 |  | MRPL39 |
| HLA-C | HLA-DP | CHMP4B | GAPDH |  | MRPL4 |
| HSPG2 | HLA-DQ | CHMP4BP1 | HSPA1A |  | MRPL40 |
| Igta1 | HLA-DR | CHMP4C | TUBA1A |  | MRPL41 |
| Igtav | ITGA2B | CHMP5 | TUBA1B |  | MRPL42 |
| ITGA1 | PECAM | CHMP6 | TUBA1C |  | MRPL43 |
| ITGA10 | PTPRC | CHMP7 | TUBA3C |  | MRPL44 |
| ITGA11 | RNASEH2A | EHD1 | TUBA3D |  | MRPL45 |
| ITGA2 | THY1 | EHD2 | TUBA3E |  | MRPL46 |
| ITGA2B | TSPAN8 | EHD3 | TUBA4A |  | MRPL47 |
| ITGA3 |  | EHD4 | TUBA4B |  | MRPL48 |
| ITGA4 |  | FLOT1 | TUBA8 |  | MRPL49 |
| ITGA5 |  | FLOT2 | TUBAL3 |  | MRPL50 |
| ITGA6 |  | HSP90AB1 | TUBB |  | MRPL51 |
| ITGA7 |  | HSPA8 | TUBB1 |  | MRPL52 |
| ITGA8 |  | MAPT | TUBB2A |  | MRPL53 |
| ITGA9 |  | PDCD6IP | TUBB2B |  | MRPL54 |
| ITGAD |  | RHOA | TUBB3 |  | MRPL55 |
| ITGAE |  | SDCBP | TUBB4A |  | MRPL57 |
| ITGAL |  | TSG101 | TUBB4B |  | MRPL9 |
| ITGAM |  | VPS4A | TUBB6 |  | MRPS10 |
| ITGAV |  | VPS4B | TUBB8 |  | MRPS11 |
| ITGAX |  |  | TUBB8B |  | MRPS12 |
| ITGB1 |  |  | TUBD1 |  | MRPS14 |
| ITGB1BP1 |  |  | TUBE1 |  | MRPS15 |
| ITGB1BP2 |  |  | TUBG1 |  | MRPS16 |
| ITGB2 |  |  | TUBG2 |  | MRPS17 |
| ITGB3 |  |  | TUBGCP2 |  | MRPS18A |
| ITGB4 |  |  | TUBGCP3 |  | MRPS18B |
| ITGB5 |  |  | TUBGCP4 |  | MRPS18C |
| ITGB6 |  |  | TUBGCP5 |  | MRPS2 |
| ITGB7 |  |  | TUBGCP6 |  | MRPS21 |
| ITGB8 |  |  |  |  | MRPS22 |
| ITGBL1 |  |  |  |  | MRPS23 |
| LAMP1 |  |  |  |  | MRPS24 |
| LAMP2 |  |  |  |  | MRPS25 |
| NT5E |  |  |  |  | MRPS26 |
| SDC1 |  |  |  |  | MRPS27 |
| SDC2 |  |  |  |  | MRPS28 |
| SDC3 |  |  |  |  | MRPS30 |
| SDC4 |  |  |  |  | MRPS31 |
| SHH |  |  |  |  | MRPS33 |
| TFR2 |  |  |  |  | MRPS34 |
|  |  |  |  |  | MRPS35 |
|  |  |  |  |  | MRPS36 |
|  |  |  |  |  | MRPS5 |
|  |  |  |  |  | MRPS6 |
|  |  |  |  |  | MRPS7 |
|  |  |  |  |  | MRPS9 |
|  |  |  |  |  | RPL10 |
|  |  |  |  |  | RPL10A |
|  |  |  |  |  | RPL11 |
|  |  |  |  |  | RPL12 |
|  |  |  |  |  | RPL13 |
|  |  |  |  |  | RPL13A |
|  |  |  |  |  | RPL14 |
|  |  |  |  |  | RPL15 |
|  |  |  |  |  | RPL17 |
|  |  |  |  |  | RPL18 |
|  |  |  |  |  | RPL18A |
|  |  |  |  |  | RPL19 |
|  |  |  |  |  | RPL21 |
|  |  |  |  |  | RPL22 |
|  |  |  |  |  | RPL23 |
|  |  |  |  |  | RPL23A |
|  |  |  |  |  | RPL24 |
|  |  |  |  |  | RPL26 |
|  |  |  |  |  | RPL27 |
|  |  |  |  |  | RPL27A |
|  |  |  |  |  | RPL28 |
|  |  |  |  |  | RPL29 |
|  |  |  |  |  | RPL3 |
|  |  |  |  |  | RPL30 |
|  |  |  |  |  | RPL31 |
|  |  |  |  |  | RPL32 |
|  |  |  |  |  | RPL34 |
|  |  |  |  |  | RPL35 |
|  |  |  |  |  | RPL35A |
|  |  |  |  |  | RPL36 |
|  |  |  |  |  | RPL36A |
|  |  |  |  |  | RPL37 |
|  |  |  |  |  | RPL37A |
|  |  |  |  |  | RPL38 |
|  |  |  |  |  | RPL39 |
|  |  |  |  |  | RPL3L |
|  |  |  |  |  | RPL4 |
|  |  |  |  |  | RPL41 |
|  |  |  |  |  | RPL5 |
|  |  |  |  |  | RPL6 |
|  |  |  |  |  | RPL7 |
|  |  |  |  |  | RPL7A |
|  |  |  |  |  | RPL8 |
|  |  |  |  |  | RPL9 |
|  |  |  |  |  | RPS10 |
|  |  |  |  |  | RPS11 |
|  |  |  |  |  | RPS12 |
|  |  |  |  |  | RPS13 |
|  |  |  |  |  | RPS14 |
|  |  |  |  |  | RPS15 |
|  |  |  |  |  | RPS15A |
|  |  |  |  |  | RPS16 |
|  |  |  |  |  | RPS17 |
|  |  |  |  |  | RPS18 |
|  |  |  |  |  | RPS19 |
|  |  |  |  |  | RPS2 |
|  |  |  |  |  | RPS20 |
|  |  |  |  |  | RPS21 |
|  |  |  |  |  | RPS23 |
|  |  |  |  |  | RPS24 |
|  |  |  |  |  | RPS25 |
|  |  |  |  |  | RPS26 |
|  |  |  |  |  | RPS27 |
|  |  |  |  |  | RPS27AP5 |
|  |  |  |  |  | RPS27L |
|  |  |  |  |  | RPS28 |
|  |  |  |  |  | RPS29 |
|  |  |  |  |  | RPS3 |
|  |  |  |  |  | RPS3A |
|  |  |  |  |  | RPS5 |
|  |  |  |  |  | RPS6 |
|  |  |  |  |  | RPS7 |
|  |  |  |  |  | RPS8 |
|  |  |  |  |  | RPS9 |
|  |  |  |  |  | RPSA |
|  |  |  |  |  | UMOD |
